# Automated and rapid detection of cancer in suspicious axillary lymph nodes in patients with breast cancer

**DOI:** 10.1038/s41523-021-00298-6

**Published:** 2021-07-07

**Authors:** Juanjuan Li, Bradley M. Downs, Leslie M. Cope, Mary Jo Fackler, Xiuyun Zhang, Chuan-gui Song, Christopher VandenBussche, Kejing Zhang, Yong Han, Yufei Liu, Suzana Tulac, Neesha Venkatesan, Timothy de Guzman, Chuang Chen, Edwin W. Lai, Jingping Yuan, Saraswati Sukumar

**Affiliations:** 1 Department of Breast and Thyroid Surgery, Renmin Hospital of Wuhan University, Wuhan, China; 2 Department of Oncology, Johns Hopkins University School of Medicine, Baltimore, MD USA; 3 Department of Pathology, Renmin Hospital of Wuhan University, Wuhan, China; 4 Department of Breast Surgery, Union Hospital Affiliated by Fujian Medical University, Fuzhou, China; 5 Department of Pathology, Johns Hopkins University School of Medicine, Baltimore, MD USA; 6 Department of Breast Surgery, Xiangya Hospital, Central South University, Changsha, China; 7 Department of Thyroid and Breast Surgery, Binzhou Medical University Hospital, Binzhou, China; 8 Department of Pathology, Yichang Central People’s Hospital, Yichang, China; 9 Oncology R&D, Cepheid, Sunnyvale, CA USA

**Keywords:** Biomarkers, Diagnostic markers

## Abstract

Preoperative staging of suspicious axillary lymph nodes (ALNs) allows patients to be triaged to ALN dissection or to sentinel lymph node biopsy (SLNB). Ultrasound-guided fine needle aspiration (FNA) and cytology of ALN is moderately sensitive but its clinical utility relies heavily on the cytologist’s experience. We proposed that the 5-h automated GeneXpert system-based prototype breast cancer detection assay (BCDA) that quantitatively measures DNA methylation in ten tumor-specific gene markers could provide a facile, accurate test for detecting cancer in FNA of enlarged lymph nodes. We validated the assay in ALN-FNA samples from a prospective study of patients (*N* = 230) undergoing SLNB. In a blinded analysis of 218 evaluable LN-FNAs from 108 malignant and 110 benign LNs by histology, BCDA displayed a sensitivity of 90.7% and specificity of 99.1%, achieving an area under the ROC curve, AUC of 0.958 (95% CI: 0.928–0.989; *P* < 0.0001). Next, we conducted a study of archival FNAs of ipsilateral palpable LNs (malignant, *N* = 72, benign, *N* = 53 by cytology) collected in the outpatient setting prior to neoadjuvant chemotherapy (NAC). Using the ROC-threshold determined in the prospective study, compared to cytology, BCDA achieved a sensitivity of 94.4% and a specificity of 92.5% with a ROC-AUC = 0.977 (95% CI: 0.953–1.000; *P* < 0.0001). Our study shows that the automated assay detects cancer in suspicious lymph nodes with a high level of accuracy within 5 h. This cancer detection assay, scalable for analysis to scores of LN FNAs, could assist in determining eligibility of patients to different treatment regimens.

## Introduction

Axillary lymph node (ALN) status is an important independent prognostic factor for early breast cancer, as it is predictive of disease-free survival and overall survival^1–3^. Accurate identification of metastasis to the ALN preoperatively is essential for staging and planning of treatment regimens, including neoadjuvant chemotherapy (NAC)^4^, postmastectomy radiation, and consideration of reconstruction^5–7^. NAC before surgery is now considered a standard step in treating node-positive breast cancer^8^. Furthermore, postmastectomy radiation is recommended for select breast cancer patients with more than one involved lymph node since it has been shown to improve disease-free survival and overall survival^9–11^. Therefore, initial axillary lymph node evaluation is clinically significant. Moreover, the standard staging of the axilla preoperatively in women presenting with clinically negative lymph nodes is important since it will allow patients to be triaged to axillary lymph node dissection with a positive test result, or to sentinel lymph node biopsy (SLNB) with a negative test result^12^. ALN dissection is an invasive procedure with a high level of morbidity and is associated with complications such as seroma, lymphedema, and nerve injury^13^ while SLNB is less risky. In fact, large clinical studies have reported that SLNB has a lower morbidity rate than axillary lymph node dissection, but similar accuracy^14–17^.

Currently, various imaging tests including ultrasound, mammography, and magnetic resonance imaging (MRI) can provide information on axillary lymph nodes (ALNs) prior to surgery that aid in staging and treatment. However, the accuracy and sensitivity of these imaging techniques are relatively low. It has been reported that positron-emission tomography (PET) or PET integrated with computed tomography (PET/CT) has a mean sensitivity of 63% and a mean specificity of 94%^18^. Furthermore, these screening techniques are costly to perform. Several studies have provided strong support that preoperative ultrasound-guided fine needle aspiration (FNA) cytology can evaluate suspicious lymph nodes with accuracy in women with breast cancer^19–21^. However, as shown by a systematic review of 12 articles (1802 patients), while cytology is highly specific [96% (95% CI, 94–98%)], it is only moderately sensitive [74% (95% CI, 72–77%)]^22^, and depends on the local surgical practice and experience of the cytologists. Thus, FNA-cytology may have limited clinical utility^21,23–25^. Therefore, a test with a high level of sensitivity and specificity for accurate determination of a suspicious lymph node as benign or positive for cancer to assist with the further staging of breast cancer is urgently needed. In addition, if the test results are obtained within a few hours of the FNA procedure, this will reduce the time to diagnosis.

DNA methylation in gene promoter regions is a molecular modification of DNA that is tightly associated with loss of gene expression^26^. Many studies have shown that promoter regions of tumor suppressor genes are commonly hypermethylated during the process of tumorigenesis^27,28^. We have previously shown that breast cancer-specific hypermethylated genes can serve as powerful markers for the detection of breast cancer in body fluids such as nipple aspiration, ductal lavage, and core biopsies^15,29–31^. We have also reported the identification of methylated gene marker panels which are specific to preinvasive and invasive ductal and lobular cancers^30,32–38^. In a recent study, we developed an automated cartridge-based prototype breast cancer detection assay (BCDA) (Research Use Only) for use on the Cepheid GeneXpert system for quantitative analysis of a panel of ten hypermethylated gene markers in FNA that distinguished between benign and malignant breast lesions with 96% sensitivity and 90% specificity (ROC-AUC: 0.960, 95% CI = 0.883–1.0)^39^.

In this study, we used BCDA^39^ on FNA of suspicious lymph nodes in patients with palpable breast lesions to determine its accuracy in detecting cancer cells in ipsilateral ALNs. We tested de-identified LN-FNA samples collected in a prospective study in the intraoperative (sentinel LN biopsy) setting and showed that the automated assay detects the presence of cancer with a high level of accuracy. In a pilot clinical validation study LN-FNA, collected from women with and without breast cancer in the outpatient setting, was analyzed by BCDA. Compared to cytology alone, BCDA achieved higher than 90% sensitivity and specificity. Based on these results, further evaluation of this automated assay in larger prospective studies of LN-FNA is warranted.

## Results

### Automated cartridge-based BCDA

**Fig. 1:**
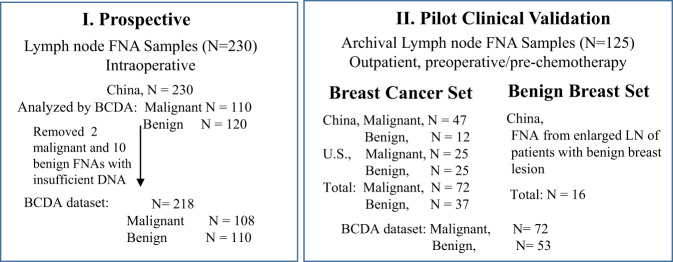
Study workflow and sample selection. Two studies were conducted sequentially, **I** The prospective study was conducted on patients with breast cancer undergoing SLNB. LN-FNA was collected, after excision, from one selected, enlarged sentinel node for cytology and BCDA. Histological diagnosis of the same LN served as a gold standard. **II** A pilot clinical validation on archival FNA. BCDA was performed on archival LN-FNA samples, selected by the cytologists. FNA was collected from an enlarged ipsilateral axillary lymph node in women with breast cancer (Breast Cancer Set) or a benign breast lesion (Benign Breast Set). Lymph nodes were aspirated for cytology in the outpatient center; in cancer patients, this occurred preoperatively or prior to initiation of neoadjuvant chemotherapy. Samples from both studies were de-identified and blinded to the lab personnel. FNA fine needle aspirate. Total FNA samples: Intraoperative prospective blinded study of *N* = 230, evaluable FNA samples: *N* = 218. By histopathology: Malignant, *N* = 108, Benign, *N* = 110; Pilot clinical validation study: *N* = 125. By cytology, Malignant, *N* = 72, Benign, *N* = 53. In the group of benign LNs, 37 LN-FNAs were from women with breast cancer, and 16 LN-FNAs were from women with benign breast disease.

We recently reported that the methylation status of a ten-gene panel tested on FNA of suspicious breast lesions by the automated prototype BCDA run on the GeneXpert system could distinguish between benign and malignant breast cancer with higher than 90% sensitivity and 90% specificity^39^. To test whether the same cartridge-based BCDA could be used to diagnose lymph node involvement in breast cancer patients, we performed a prospective and pilot clinical validation assay using FNA samples. The study design for both studies is shown in Fig. 1.

#### Verification of the automated assay in LN-FNAs from the prospective study

**Fig. 2:**
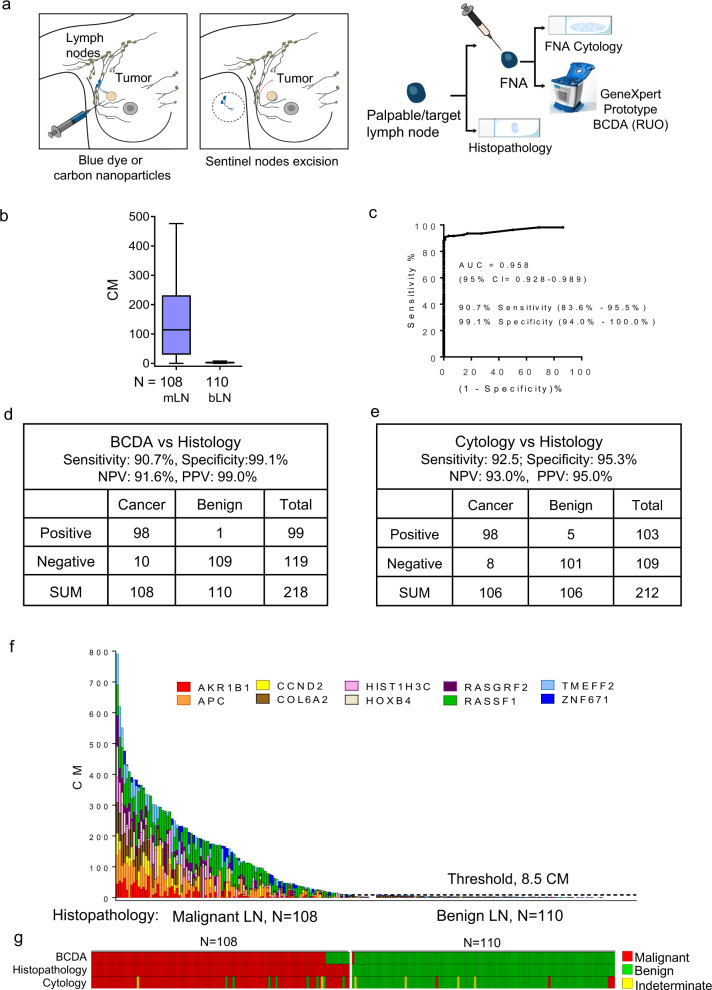
Performance of the 10-gene methylated marker panel in detecting positive lymph nodes in women with breast cancer using the GeneXpert Prototype BCDA. FNA was collected from lymph nodes intraoperatively during SLNB for cytology and molecular analysis prior to assessment by histology. **a** FNA collection schema of the prospective study sample set. **b** Box plots show the median cumulative methylation of FNA from malignant lymph nodes versus benign lymph nodes by histology. For the box plot, the whiskers are Tukey, the box is 25th and 75th percentiles, and the center bar is the median. **c** Receiver operating characteristic (ROC) analysis of the area under the curve (AUC) indicates the discriminatory power of the 10-gene marker panel at a threshold for benign at 8.5 CM units. **d** Performance of BCDA compared to the gold standard of histopathology of the sentinel lymph node removed at surgery. NPV and PPV of BCDA are presented. **e** Performance of cytology compared to histopathology. For cytology comparisons, two FNA samples of malignant lymph node and four samples of benign lymph node which were of indeterminate cytology (yellow bars) were removed from the analysis. NPV and PPV of cytology are presented. **f** Histogram plots indicate the percent methylation (colored segment) in each gene and cumulative methylation of the 10-gene marker panel by BCDA in each patient sample. **g** A heat map of LN-FNA samples positive (red) and negative (green) for methylation by BCDA compared to histopathology, and cytology. Yellow bars: indeterminate by cytology. BCDA breast cancer detection assay, CM cumulative methylation units, NPV negative predictive value, PPV positive predictive value, RUO research use only.

BCDA was tested in samples from a prospective study of FNAs (*N* = 230) collected intraoperatively in an SLNB setting (Fig. 1). The clinical characteristics of patients in this prospective set are shown in Table 1 and the study design and FNA collection schema are shown in Figs 1 and 2a. Of the 230 LN-FNAs, 218 (malignant, *N* = 108, benign *N* = 110 by histology) yielded sufficient material for both cytology and BCDA. Percent methylation of each gene in the de-identified FNA samples was computed by interpolating on a standard curve of methylation in mixtures of cell line DNA ranging from 3.12 to 100% methylation (Supplementary Fig. 1a). Analysis of percent methylation in the samples, assessed for each gene in this sample set, showed a highly significant difference in methylation levels (Mann–Whitney *P* values ranging from 0.0001 to 0.0057) between malignant and benign LN-FNAs (Supplementary Fig. 1b).

**Table 1:** Patient characteristics in the prospective study.

Patient characteristics	Case-control set	
Region (*N*)	China (*N* = 218)	
Summary	Case (*N* = 108)	Control (*N* = 110)
***Invasive ductal carcinoma***	*N* = 95	*N* = 98
**Age in years, Median (Range)**	49.5 (31–79)	52 (30–80)
**Receptor status:**		
ER/PR+, HER2−	37	51
ER/PR+, HER2+	29	8
ER/PR−, HER2+	13	15
ER/PR−, HER2−	11	14
Unknown	5	10
**Stage (AJCC 8th):**		
I	0	36
II	37	51
III	50	4
IV	0	0
Unknown	8	7
***Other invasive carcinoma***	*N* = 13	*N* = 12
**Age (in years), Median (range)**	52 (43–72)	56.5 (39–65)
**Receptor status:**		
ER/PR+, HER2−	6	7
ER/PR+, HER2+	3	2
ER/PR−, HER2+	2	0
ER/PR−, HER2−	2	3
Unknown	0	0
**Stage (AJCC 8th):**		
I	0	3
II	4	7
III	8	2
IV	0	0
Unknown	1	0

Cumulative methylation (CM), or sum of methylation of the ten gene-panel, was high in the majority of the malignant lymph nodes and low or absent in the benign. Analysis of the CM of all ten markers in the two groups, presented in the box plot, shows a significant difference in methylation between malignant and benign LNs (*P* < 0.0001; Fig. 2b). Receiver operating characteristics analysis showed that, compared to histopathology, the methylated biomarker panel displayed an area under the ROC curve, AUC = 0.958 (95% CI: 0.928–0.989) (Fig. 2c). We compared the sensitivity and specificity of BCDA (at a threshold of 8.5 CM) to the gold standard of histopathology of the sentinel lymph node, and to FNA cytology. Compared to histopathology, BCDA achieved a negative predictive value (NPV) of 91.6%, and a positive predictive value (PPV) of 99.0% (Fig. 2d). Compared to histopathology, cytology achieved a sensitivity of 92.5% (95% CI: 85.6–96.6%) and specificity of 95.3% (95% CI: 89.33−98.45%), and an NPV of 93.0%, and a PPV of 95.0% (Fig. 2e). The histogram in Fig. 2f shows the percent methylation in each gene in the panel in LN-FNA from each patient and highlights the significant difference in CM of the ten genes in malignant versus benign FNAs. The heatmap in Fig. 2f showed a high level of concordance of calls between BCDA to histopathology of the same sentinel lymph node and to cytology of the FNA.

We examined the potential advantage of combining the two assays, BCDA and cytology, for more precise detection of tumor cells in the LN. Used together, the assays achieve a sensitivity of 99.1% (95% CI: 94.1–99.5%), with a specificity of 94.6% (95% CI: 88.5–97.9%) resulting in an improvement in sensitivity; but with some loss of specificity.

To compare the performance of BCDA with the well-established laboratory assay, quantitative multiplex methylation-specific PCR (QM-MSP), we analyzed the methylation status of a subset of FNA from malignant (*N* = 73) and benign (*N* = 87) LNs from the prospective study by this method. Cumulative methylation index (CMI) as assessed by QM-MSP for the 10-gene panel was higher in malignant LN compared to benign LN as shown in the histogram (Supplementary Fig. 2a) and in the box-plot (Supplementary Fig. 2b). ROC analysis established the laboratory threshold (CMI of 13.5) that provided the optimal sensitivity, while retaining ≥90% specificity (Supplementary Fig. 2c). Like BCDA, QM-MSP yielded a high level of sensitivity of 91.8% and a specificity of 97.7% and a ROC-AUC = 0.966 (95% CI: 0.934–0.998) (Supplementary Fig. 2c). CM measurements of the ten markers using the two assays, QM-MSP and the automated cartridge assay, were highly correlated with a Spearman correlation of *r* = 0.874 (Supplementary Fig. 2d).

#### Pilot clinical validation of BCDA on LN-FNA samples

**Fig. 3:**
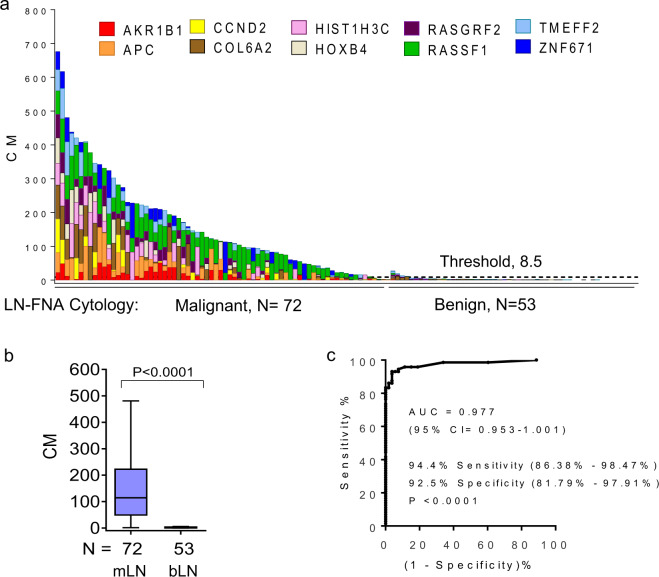
Pilot clinical validation of the automated BCDA on FNA samples collected prior to neoadjuvant chemotherapy. The results of BCDA were compared to cytology-based diagnosis of the FNA. **a** Histogram shows the 10-gene marker panel distinguished between malignant (*N* = 72) and benign (*N* = 53) FNA samples collected in the outpatient clinic using a threshold set for benign at 8.5 CM based on the prospective study (Fig. 2). **b** Box plot shows that cumulative methylation is significantly higher in malignant compared to benign breast LN-FNA. Mann–Whitney *P* values are shown. For the box plot, the whiskers are Tukey, the box is 25th and 75th percentiles, and the center bar is the median. **c** ROC: AUC analysis shows the discriminatory power of the 10-gene marker panel. BCDA performed with a sensitivity of 94.4%, and a specificity of 92.5%, and ROC, AUC: 0.977 (95% CI: 0.953–1.00). FNA fine needle aspiration, *N* number of samples.

FNA samples from suspicious, enlarged lymph nodes were collected under ultrasound guidance in China and United States, and the archival stained slides were analyzed in a blinded manner in the automated BCDA. Clinical characteristics of the patients in this study are presented in Table 2, and the study design is presented in Fig. 1. From women with breast cancer, the cytologists at the respective institutions in China and the United States selected archival malignant (*N* = 72) and benign (*N* = 37) lymph node FNAs (Fig. 1). In addition, 16 benign FNA samples from enlarged lymph nodes in women with benign breast lesions were included. Thus, a total of 72 malignant and 53 benign lymph node FNAs were analyzed by BCDA. CM of the 10-gene panel by BCDA of the LN-FNA is shown in the histogram in Fig. 3a. The difference in CM in malignant and benign FNA samples was highly significant (*P* < 0.0001, Fig. 3b). In this set, results of BCDA were compared to cytology since no core biopsy for histology was performed on the lymph nodes prior to initiation of neoadjuvant therapy. Compared to cytology, BCDA achieved a sensitivity of 94.4% and a specificity of 92.5% [ROC; AUC = 0.977 (95% CI: 0.953–1.001)] using a ROC threshold of 8.5 CM derived in the previous study (Fig. 3c).

**Table 2:** Patient characteristics in the pilot clinical validation study.

Patient characteristics	Pilot validation set			
Region	United States		China	
**LN FNA (Breast cancer)**	Malignant *N* = 25	Benign *N* = 25	Malignant *N* = 47	Benign *N* = 28
***Invasive ductal carcinoma***	*N* = 25	*N* = 25	*N* = 43	*N* = 11
**Age in years, median (range)**	59 (28–80)	52 (24–89)	49 (41–67)	53 (47–68)
**Receptor status:**				
ER/PR+, HER2−	13	8	14	2
ER/PR+, HER2+	4	4	16	6
ER/PR−, HER2+	5	1	6	1
ER/PR−, HER2−	2	10	6	2
Unknown	1	2	1	0
**Stage (AJCC 8th):**				
I	1	13	7	26
II	12	10	7	31
III	12	0	15	52
IV	0	0	2	3
Unknown	0	0	12	0
***Other invasive carcinoma***	0	0	4	1
**Age (in years), median (range)**	–	–	52 (46–55)	55
**Receptor status:**				
ER/PR+, HER2−	–	–	2	0
ER/PR+, HER2+	–	–	1	1
ER/PR−, HER2+	–	–	0	0
ER/PR−, HER2−	–	–	0	0
Unknown	–	–	1	0
**Stage (AJCC 8th):**				
I	–	–	0	1
II	–	–	0	0
III	–	–	3	0
IV	–	–	0	0
Unknown	–	–	1	0
**LN FNA (Benign breast disease)**	0	0	0	16
**Age in years, median (range)**	–	–	–	32.5 (25–60)

### Subgroup analysis on DNA methylation in FNA samples of lymph nodes

#### Age

In this study, we found that the age of the patients with benign disease in the LN was matched with the age of the patients with malignant disease (Supplementary Fig. 3a). A linear regression model of CM fit on benign and malignant LNs showed a nonsignificant increase in methylation with greater age for malignant lymph nodes (*P* = 0.166, *r* = 0.032; Supplementary Fig. 3b). To understand how age affects the classification of the benign samples, logistic regression models were constructed for multiple thresholds of methylation. The results on pooled data from samples in the prospective and pilot clinical validation study indicate that at lower CM thresholds, older patients with the benign disease are more likely to be misclassified (Supplementary Fig. 3c). Furthermore, in older patients, tumor samples have a decreased risk of false negatives (Supplementary Fig. 3d).

#### IHC subtype of primary breast cancer

We used a subset of data on CM from LN-FNA from both the prospective and pilot clinical validation studies (*N* = 172) for whom IHC subtype data was available. Using a laboratory threshold value of 8.5 CM units for benign, malignant lymph nodes in primary breast cancer patients showed a nonsignificant difference in call rate between the four subtypes^40^, ER/PR^+^, HER2^−^; ER/PR^+^, HER2^+^; ER/PR^−^, HER2^+^; and ER/PR^−^, HER2^−^ (triple-negative breast cancer, TNBC) (Fisher’s Exact *P* = 0.622, Supplementary Table 2a). However, CM of the 10-marker panel (Supplementary Fig. 4a), and median percent methylation for each of 7/10 genes in the panel showed a significant difference (*P* = 0.001 to 0.0002, Kruskal–Wallis) between subtypes (Supplementary Table 2b and Supplementary Fig. 4b). Between the four subtypes, individual markers showed varying extent of methylation. AKR1B1, APC, COL6A2, and HOXB4 were frequently more methylated (<0.001) in ER/PR^+^, HER2^−^ tumors, while APC and RASSF1 were frequently methylated (*p* < 0.0001) in HER2-overexpressing tumors (Supplementary Table 2b and Supplementary Fig. 4b).

#### Region

Performance of the 10-gene panel (as measured by area under the ROC curve) was assessed for LN-FNAs from the United States and China, in both prospective and pilot clinical validation studies. No significant regional differences were seen, since, as shown in Supplementary Fig. 5a, b, BCDA achieved a sensitivity of 88.0% and a specificity of 92.0% [ROC; AUC = 0.982 (95% CI: 0.956–1.009)] for samples from the United States and a sensitivity of 97.8% and a specificity of 92.9% (ROC; AUC = 0.964 (95% CI: 0.940–0.988) for samples from China. However, the lower level of detection sensitivity of 88.0% in the LN-FNA samples from the United States compared to 97.8% in samples from China (Supplementary Fig. 5b), prompted us to examine some factors that might have contributed to this discrepancy. We found that there was no significant difference in CM in the malignant LN-FNAs in samples from either country (*P* = 0.882). A modest but significant difference was observed between the benign LN-FNAs (*P* = 0.035) from the two countries. However, it should be noted that very low levels of methylation were observed in benign LN-FNAs (China: median CM 2.4; U.S: median CM 1.4) (Supplementary Fig. 6a, b), and at these low levels, results can vary between assays. No difference in methylation was observed for the various immunohistochemical subtypes of breast cancer (Supplementary Fig. 6c–f). Study participants from China with malignant LNs were slightly younger than those from the United States, but did not reach statistical significance (*P* = 0.057), while there was no significant difference in age of the patients with benign LN-FNA (Supplementary Fig. 6g, h). Lastly, the length of storage of the LN-FNA samples was investigated. Here, for both malignant and benign samples, the samples from the United States were significantly older and were collected from years 2012–2016, while samples from China were from 2016–2019 (Supplementary Fig. 6i, j). This observation raises the possibility that the age of the slides may have contributed to lower levels of methylation detection, but the evidence is not conclusive.

#### Stage of the primary tumor and nodal status

We investigated if there was a correlation between cumulative and single gene methylation in FNA of malignant lymph node and the clinical stage of breast cancer in a subset of 72 of 108 cases in the prospective study. CM of 10-marker panel or single gene methylation in FNA of malignant LNs in patients with stage III and IV breast cancer showed no difference when compared to malignant LNs in patients with stage I/II breast cancer (Mann–Whitney statistics, *P* = 0.755; Supplementary Fig. 7a, b). CM or single gene methylation was also not different in patients who had 1–3 positive nodes compared to patients with 4 or more positive nodes (*P* = 0.125, Supplementary Fig. 7c, d). One exception was that the LN-FNA methylation of APC in patients with 4 or more positive nodes was higher than in patients with 1–3 positive nodes (*P* = 0.021, Mann–Whitney test; Supplementary Fig. 7d).

#### Response to NAC

In the pilot clinical validation cohort, 46.4% (58/125) patients from China and the United States received NAC. For this exploratory subgroup analysis, LN-FNA data on 49 out of the 58 patients with available treatment response information following NAC were examined. Assessment of pathological response to NAC was done using the Miller–Payne test. Response to NAC in this study was defined as near-complete and complete responder group (Miller–Payne grades 4 and 5). CM of the 10-gene panel in malignant lymph nodes or single gene comparisons showed no difference between patients with a positive response versus no-response to NAC (*P* = 0.971, Mann–Whitney; Supplementary Fig. 7e, f).

In summary, we have extended the utility of the automated BCDA to detecting cancer in suspicious lymph nodes. We have piloted a 5-h test to detect cancer in FNA of suspicious LNs collected in the outpatient setting. This test has the potential to determine axillary involvement with a higher sensitivity than cytology within a very short time in the outpatient setting.

## Discussion

In the current study, we showed that the previously described 10-marker panel that can distinguish breast cancer from normal/benign breast tissues can also be used to distinguish between lymph nodes positive for breast cancer cells from normal/benign lymph nodes with high sensitivity and specificity. Furthermore, we also confirmed that the automated prototype BCDA run on a GeneXpert system was highly effective in detecting cancer in FNA of suspicious LNs.

With histopathology of the same lymph node as the gold standard, the methylated biomarker panel displayed a sensitivity of 90.7% and a specificity of 99.1% (ROC; AUC = 0.958, 95% CI: 0.928–0.989). Cytology of the palpable enlarged LNs achieved a slightly higher cancer detection sensitivity of 92.5% (85.67% − 96.69%) versus 90.7% by BCDA, and lower specificity of 95.3% (89.33–98.45%) compared to 99.1% by BCDA (Fig. 2). With these results, we have demonstrated higher than 90% sensitivity/specificity of a panel of ten hypermethylated biomarkers to distinguish between malignant and benign lymph node from breast cancer patients. Together, BCDA and cytology achieved a sensitivity of 99.1% (95% CI: 0.941–0.995), with a specificity of 94.6% (95% CI: 88.51–97.97). Thus, sensitivity improves considerably by combining the two assays, but this occurred at the expense of the high specificity achieved by BCDA alone. Since a high level of sensitivity of detection is important in the staging of cancer, the combined use of the two assays may be beneficial. Subsequent core biopsy or intraoperative SLNB could triage cases with false-positive lymph nodes.

Age-related methylation patterns have recently been reported in independent sample sets and between different populations^41,42^. The DNA methylome appears to undergo extensive epigenetic remodeling resulting in modulation of multiple biological pathways, particularly those that are related to aging and age-related diseases. Therefore, we performed an exploratory analysis of age-associated DNA methylation changes in our panel of ten genes in all LN-FNA samples (Supplementary Fig. 3). A linear regression model of CM by BCDA as a function of age, fit on benign/normal LN samples demonstrated a modest, non-statistically significant increase in methylation with greater age. This was consistent with our previous studies^30,32,39^.

We found significant differences in CM of the 10-gene marker panel by IHC subtype of the primary BC, indicating that our marker panel may predict detailed characteristics about the initial diagnosis of HER2-positive cancer regardless of the status of hormone receptors (Supplementary Table 2 and Supplementary Fig. 4). Some genes in the panel, such as AKR1B1, APC, COL6A2, and HOXB4 were frequently methylated in ER/PR+, HER2− tumors^34,43^, while APC and RASSF1 were frequently methylated in HER2 over-expressed tumors, which was also consistent with the previous reports^34,44^.

Our study has several strengths and a few limitations. We have developed an automated DNA methylation-based assay to detect cancer cells in FNAs of lymph nodes (Fig. 3), with an accuracy equivalent to that of our highly sensitive but labor-intensive laboratory assay, QM-MSP (Supplementary Fig. 2). QM-MSP is set up to analyze up to 24 patient samples at a time, but from start to finish the method involves a CLIA level lab (or research lab), and a minimum of 2 weeks to process 10-genes. In contrast, BCDA can evaluate from one to hundreds of patient samples at a time, in “real-time” of 5 h and can be performed in a routine clinical lab. The machine is modular and linkable to other modules driven by a single computer interface. Samples can be analyzed in a single module, one at a time, or in multiples of 1–16 modules at the same time. Each cartridge is powered by its own PCR machinery—the barcode on it determines the conditions of the experiment for that cartridge. A further advantage of BCDA is that it is sophisticated but simple to operate, and therefore easy to implement in less-resourced settings. Thousands of GeneXpert^®^ machines are available in both low- and high-income countries due to the popularity of TB testing and a wide array of tests for bacterial and viral infections, rendering the assay affordable with minimal additional investment.

This test has the potential to determine axillary involvement with higher sensitivity than cytology within a very short time in the outpatient setting. Although the prospective study was performed in the SLNB setting, we are not envisioning the use of this assay for intraoperative analysis of SLNs. That study design was utilized to enable validation of BCDA using histopathology as a gold standard in addition to cytology. Our proposed use for the assay is in the outpatient clinic to analyze enlarged LNs, which are frequently tumor-free. However, the presence of palpable LNs renders treatment choices more difficult in those patients. Also, in patients selected for NAC, it is important to distinguish whether the palpable LN contains disseminated tumor cells or whether LN enlargement is a consequence of a cancer-unrelated event. In these cases, a 5-h test with the results provided by the end of the day will, potentially, be very useful.

A positive test preoperatively could provide guidance for selecting patients for neoadjuvant treatment, radiation after mastectomy, and to ensure clear lymph nodes prior to breast reconstruction. High specificity in distinguishing between malignant and benign lymph node was found in both prospective and validation sets. This would allow early breast cancer patients without lymph node metastasis to avoid unnecessary axillary lymph node dissection, which involves a high risk of complications such as seroma, lymphedema, and nerve injury^13^. Additionally, BCDA is a simplified, quick, and automated assay where evaluation and analysis are standardized to minimize operator errors.

There are several limitations of our study. A key limitation is the validation sets were small; the prospective intraoperative study enrolled 230 patients and pilot clinical validation in the outpatient setting was on LN FNAs from 125 patients. Further validation of BCDA in prospective blinded clinical trials of FNA samples of the lymph node in various clinical settings is necessary to confirm the accuracy of this assay. In addition, archival FNA samples in the pilot clinical validation set were collected before NAC in our clinical practice. Therefore, no histology of the same lymph node before NAC was available as the gold standard for comparison. Furthermore, our population was diverse and heterogeneous in terms of disease states, which limits the power to analyze each relevant clinical subgroup.

In conclusion, BCDA performs with accuracy and has the potential to determine axillary involvement with higher sensitivity than cytology within a very short time in the outpatient setting. Further evaluation of this automated assay in larger, blinded, prospective studies is warranted.

## Methods

### Lymph node FNA collection

The accuracy of BCDA for the identification of cancer-positive lymph nodes in breast cancer patients was evaluated in two clinical studies (Supplementary Table 1). A prospective intraoperative study was performed on FNA of a sentinel node concurrent with a biopsy to provide an opportunity for comparison of the results to the gold standard of histopathology and to cytology. A second validation study evaluated the performance of the assay on FNA collected in an outpatient setting from patients prior to a neoadjuvant trial and compared the results to cytology.

#### Prospective intraoperative study

A prospective study was conducted in China in breast cancer patients with palpable LNs (*N* = 230) who were undergoing SLNB. To do SLNB, a blue dye or carbon nanoparticles was injected near the tumor, and a small incision was made in the axillary region to trace the stained lymph vessels and to identify stained lymph nodes. Next, all the stained lymph nodes were surgically excised. A single enlarged lymph node was selected by the surgeon as the target lymph node. The target lymph node was subjected to FNA (for cytology and BCDA analysis), and submitted for diagnostic histopathology. Giemsa-stained LN-FNA slides from all 230 women were de-identified in the order of their collection and sent for BCDA analysis. We calculated sensitivity, specificity, and area under the receiver operating characteristic curve (ROC-AUC) for the marker panel by BCDA, and compared its performance to histopathology and to the cytology of the same LN.

To compare the performance of BCDA with a well-established reference assay, we also analyzed LN-FNA from a subset of patients in this study with additionally available slides (malignant LN: *N* = 73; benign: *N* = 87) by QM-MSP^30,37^.

#### Sample size

Sample size requirements were calculated by simulation, to control the width of the confidence interval for specificity, when sensitivity is fixed at 90%. The calculation was carried out using a nonparametric bootstrap analysis in which we resampled pilot data [FNA samples from primary tumors^39^] to achieve a range of sample sizes, calculating the width of the 90% confidence interval for each. A sample size of *N* = 100 per group ensures that the half-width of the 90% confidence interval is less than 0.1. Historically 50% of sentinel lymph node biopsies at the different centers in China participating in this study are malignant. Thus, we enrolled 230 women for a 95% probability of getting at least 100 in each arm.

#### The pilot clinical validation study

This study was conducted on archival FNAs collected preoperatively under ultrasound guidance from ipsilateral enlarged axillary LNs in the outpatient center. In patients with invasive breast cancer, this occurred preoperatively or prior to initiation of NAC. Based on cytologic diagnosis the study pathologists selected LN-FNA from a Breast Cancer Set consisting of 72 women (47 from China, 25 from the United States) with malignant lymph nodes, and 37 women (12 from China, 25 from the United States) with benign lymph nodes. An additional set, designated the Benign Breast Set, consisted of LN FNA from women (16 from China) with benign breast disease. Thus, a total of 72 malignant and 53 benign LN-FNAs were tested by BCDA. The FNA samples were confirmed by the cytologists to contain at least 100 cells/slide. All FNA slides from China for BCDA were stained with Giemsa, while FNA slides from the United States were stained with DiffQuik and mounted with a coverslip. No core biopsy for histology was performed on the enlarged lymph node prior to treatment. This design allowed us to determine the agreement between cytology and BCDA-based diagnosis in a clinically relevant outpatient setting.

#### Sample size

The use of a predetermined diagnostic threshold (from the prospective study described above) reduces the sample size requirement. For sensitivity/specificity above 90%, a sample size of at least 50 subjects per group ensures that the half-width of the 90% confidence interval is less than 0.1.

#### Institutional approvals

The study, performed on fixed and stained FNAs of lymph nodes from the prospective and pilot clinical validation study was approved by institutional review boards of Renmin Hospital of Wuhan University (WDRY2018-K062, PI: C. Chen), China, and Johns Hopkins, USA (JH-IRB 00047309, PI: S Sukumar). The study was conducted in accordance with the Declaration of Helsinki and the U.S. Common Rule. Patients provided informed consent for use of excess cells and tissue for research.

### Sample-inclusion/exclusion criteria

Stained slides of FNA were obtained from one palpable sentinel lymph node (SLN) from each patient in the prospective study (*N* = 230; 110 malignant and 120 benign), and one palpable ipsilateral lymph node in the pilot clinical validation set (*N* = 125; 72 malignant and 53 benign). The FNA slides were confirmed as containing at least 100 cells. Twelve FNA specimens (2 malignant and 10 benign) in the prospective study were discarded from the analysis due to inadequate DNA. Specimens were obtained from Renmin Hospital of Wuhan University, China following review by two pathologists and cytologists at Renmin Hospital to confirm correct classification, and from Johns Hopkins Surgical Pathology following review by one cytologist. Inclusion criteria were as follows: women of any age, histopathological diagnosis of breast cancer, and ipsilateral suspicious, palpable ALNs. Primary breast cancer included invasive ductal carcinoma (IDC), invasive lobular carcinoma (ILC), mixed invasive carcinoma, and ductal carcinoma in situ (DCIS). LN-FNA samples from a non-cancer group were also included in the study. These were samples from patients who had benign breast disease with an enlarged lymph node. The exclusion criteria were as follows: A previous or concomitant malignancy, previous systemic therapy for breast cancer, cancer chemoprevention treatment in the preceding year, Paget’s disease without invasive cancer, pregnancy, or lactation.

### The prototype BCDA for assessing methylation in lymph node FNAs

BCDA is a cartridge-based PCR assay designed to be run on the GeneXpert system (Cepheid, Sunnyvale CA)^39^. It is a prototype in development, not for use in diagnostic procedures, and has not been reviewed by any regulatory body. The assay utilizes three single-use cartridges, one of which contains reagents required for the bisulfite treatment of DNA (Cartridge A). Two additional cartridges contain reagents for quantitative PCR of Marker Set 1 and Marker Set 2 (Cartridges B, C), respectively. Each marker set consists of five target genes and ACTB as the internal reference and utilizes six fluorophores for signal detection^39^.

#### Preparation of DNA

Stained and mounted FNA slides were soaked in xylene to remove the coverslip prior to scraping and digestion. If the slide was stained but not mounted, no preprocessing was performed. The cells were scraped off the slide into proteinase K/FFPE lysis buffer solution (20 ul of proteinase K and 1.2 mL lysis buffer; FFPE Lysis Kit, 900–0697, Cepheid), digested at 80 °C for 30 min, mixed with 1.2 mL of ethanol and loaded into the bisulfite conversion Cartridge A. Upon completion of the reaction (2 h), bisulfite-treated DNA was transferred to Cartridge B and Cartridge C to quantitate gene methylation^39^ (2.5 h). One FNA slide was used for each BCDA analysis.

#### Quantitation of DNA methylation

Ct values were obtained using BCDA software for methylated targets and ACTB reference (Ct = the cycle threshold at which signal fluorescence exceeds background). For calculating percent (%) methylation, the ∆Ct (Ct Gene-Ct ACTB) value of each target gene was extrapolated from historical standard curves of mixtures of methylated and unmethylated DNA ranging from 100 to 3.12% methylation. This enabled quantitation of CM, which is the sum of percent methylation for all ten genes in the marker panel^39^.

#### Inter-platform comparison

We compared the CMI obtained by the reference laboratory assay, QM-MSP, to CM for the same ten-gene panel obtained using BCDA. For this analysis, we used a subset of samples from the prospective study that had additional slides available (malignant LN = 73 and benign LN = 87). A single LN-FNA slide was used for each assay. The R function corr.test was used to calculate the Spearman correlation.

### Differences in DNA methylation-based on immunohistochemical (IHC) subtype of the primary breast cancer

Immunohistochemical staining of ER, PR, and HER2 was performed using a standard Envision complex method. Formalin-fixed, paraffin-embedded tissue samples were cut at 4 μm, preheated at 60 °C for 1 h, and then deparaffinized and rehydrated endogenous peroxidase activity was blocked by using 3% H_2_O_2_. Antigen retrieval was carried out by microwave heating with citrate buffer (pH 6.0) for 20 min. After that, sections were incubated with primary antibody (anti-Estrogen Receptor (SP1), 790–4325, Ventana, USA; anti-Progesterone Receptor (1E2), 790–4296, Ventana, USA; anti-HER2/neu(4B5), 790–4493, Ventana, USA) for 1 h at 37 °C, and then incubated with biotinylated secondary antibody using the Dako Cytomation LSAB2 System-HRP (K0672, DakoCytomation, Carpinteria, CA, USA) for 40 min at 37 °C. After then, the sections were immersed in 3,3′diaminobenzidine (DAB) at room temperature without light for 2 or 3 min. Finally, samples were slightly counterstained with hematoxylin for 2 min. The sections with PBS, replacing the primary antibody, were used as negative controls.

We investigated if there were differences in DNA methylation as assessed by BCDA of LN-FNA samples in the four IHC subtypes, ER/PR^+^ HER2^−^; ER/PR^+^ HER2^+^; ER/PR^−^ HER2^+^; and ER/PR^−^ HER2^−^ (TNBC)^40^ of the corresponding primary breast cancer in the prospective and pilot clinical validation studies (Tables 1, 2). Samples with available subtype information from the prospective study (*N* = 99/108) and the pilot clinical validation study (*N* = 58; 34/47 from China, 24/25 from the United States) were pooled (total *N* = 172) for this analysis.

### Differences in DNA methylation-based on clinical and other characteristics

#### Patient age and geographic region

We investigated whether CM of our marker panel was associated with patient age in both studies (*N* = 180 malignant and 156 benign) and geographic region (China: 293 and United States: 50). Samples included from both prospective and pilot clinical validation studies.

#### Breast cancer stage and response to neoadjuvant chemotherapy (NAC)

We examined the correlation of CM of our marker panel and single gene methylation with clinical stage of disease (*N* = 158), number of positive lymph nodes in the patient (subset of *N* = 72/108 in the prospective study), and in the pilot clinical validation study, the response of the patient to NAC (subset of *N* = 49/58 from China and the United States who received NAC).

The Kruskal–Wallis test was used for multiple group comparisons, and the Mann–Whitney test was used for comparing two groups. The Fisher’s Exact test was performed to evaluate the differences in frequency of samples that tested positive (higher than the ROC-CM threshold) between the IHC subtypes. For the 10-gene panel, sensitivity, specificity, and ROC-AUC were evaluated for tumors from each subtype.

### Reporting summary

Further information on research design is available in the Nature Research Reporting Summary linked to this article.

## Supplementary information

Supplementary Information

Reporting Summary

## Data Availability

The data generated and analyzed during this study are described in the following data record: 10.6084/m9.figshare.14710500^45^. The following files contain the raw data associated with BCDA and QM-MSP in Figs 2, 3, and Supplementary Figs 1, 2, 3, 4, 5, 6, 7: “Figure2_Data.xlsx”, “Figure3_Data.xlsx” and “Sup_Table_Figures.xlsx”. The raw Cts (cycle threshold at which signal fluorescence exceeds background) of the method to quantitatively measures DNA methylation is in the file “Cepheid_lymphnode_Project.pdf”. None of the above files are publicly available as they contain information that could be used to identify patients and informed consent to share participant-level data was not obtained prior to or during data collection. However, they can be made available upon request, and data-related requests and enquiries should be directed to the corresponding author.

## Code availability

Code available on request from the authors.
