# Supplementary material for: Automated and rapid detection of cancer in suspicious axillary lymph nodes in patients with breast cancer
Source: NPJ Breast Cancer. 2021 Jul 7;7:89. doi: 10.1038/s41523-021-00298-6 (PMC8263765; doi:10.1038/s41523-021-00298-6)
Supplement: Supplementary file 1 — Supplementary Information [file 41523_2021_298_MOESM1_ESM.pdf]

Supplementary Table 1. General information on the study samples

	**Prospective Study set**	**Pilot Validation set**
Pathological diagnosis of breast	Breast cancer	Breast cancer or Breast benign lesion
Timing of FNA	Intraoperative, FNA on enlarged, excised sentinel LN	Preoperative or pre-chemotherapy (outpatient center)
Target lymph node	Palpable ipsilateral axillary lymph node	Enlarged ipsilateral axillary lymph node by ultrasound
Gold standard for comparison	Histopathology on the same LN	Cytopathology on the same LN

Supplementary Table 2a

BCDA: Frequency of Methylation in Malignant LN by ER/PR/HER2 Subtype of Primary Breast Cancer

Histologic Subtype	Methylated*	Not Methylated	% Methylated	P**
ER/PR+, HER2-	63	8	88.7	0.622
ER/PR+, HER2+	51	3	94.4	
ER/PR-,HER2+	25	1	96.2	
ER/PR-HER2 (TNBC)	19	2	90.5	

* Data of samples from both case-control and pilot validation studies above the threshold of 8.5 cumulative methylation (CM) units.

** Fisher's Exact test was used to compare frequency of methylation in malignant lymph node FNAs from patients with primary tumors of different histologic subtypes. No statistical significance was observed (p=0.622).

Abbreviations: TNBC, triple negative breast cancer; HER2, human epidermal growth factor receptor 2; ER, estrogen receptor; PR, progesterone receptor. LN: lymph node

Supplementary Table 2b

BCDA: Gene methylation of malignant LN by ER/PR/HER2 Subtype of Primary Breast Cancer

Histologic Subtype	Median Percent Methylation (range)									
	AKR1B1	APC	CCND2	COLA6A2	HIST1H3C	HOXB4	RASGRF2	RASSF1	TMEFF2	ZNF671
ER/PR+, HER2-	8(0-70)	27(0-100)	0(0-100)	4(0-100)	0(0-100)	6(0-100)	13(0-100)	66(0-100)	1(0-100)	0(0-81)
ER/PR+, HER2+	0(0-39)	0(0-27)	0(0-50)	1(0-69)	0(0-52)	0(0-26)	0(0-38)	20(0-66)	1(0-90)	0(0-47)
ER/PR-HER2+	6(0-63)	16(0-100)	0(0-71)	3(0-100)	0(0-100)	0(0-100)	4(0-100)	42(0-100)	3(0-100)	4(0-92)
ER/PR-HER2-TNBC	0(0-51)	0(0-33)	0(0-100)	1(0-56)	0(0-83)	0(0-45)	0(0-33)	2(0-75)	1(0-75)	0(0-100)
P*	0.0002	<0.0001	0.604	0.001	0.0001	0.0002	<0.0001	<0.0001	0.132	0.120

*Kruskal-Wallis test was used for the comparison of median percent methylation in single genes in malignant lymph node FNAs from patients with different histologic subtypes of primary breast cancer. ER/PR+/HER2-(N=71); ER/PR+/HER2+ (N54); ER/PR-/HER2+ (N=26); ER/PR-/HER2- (N=21). Median percent methylation and range is given for each individual gene.

Abbreviations: TNBC, triple negative breast cancer; HER2, human epidermal growth factor receptor 2; ER, estrogen receptor; PR, progesterone receptor.

This table accompanies Supplementary Figure S5

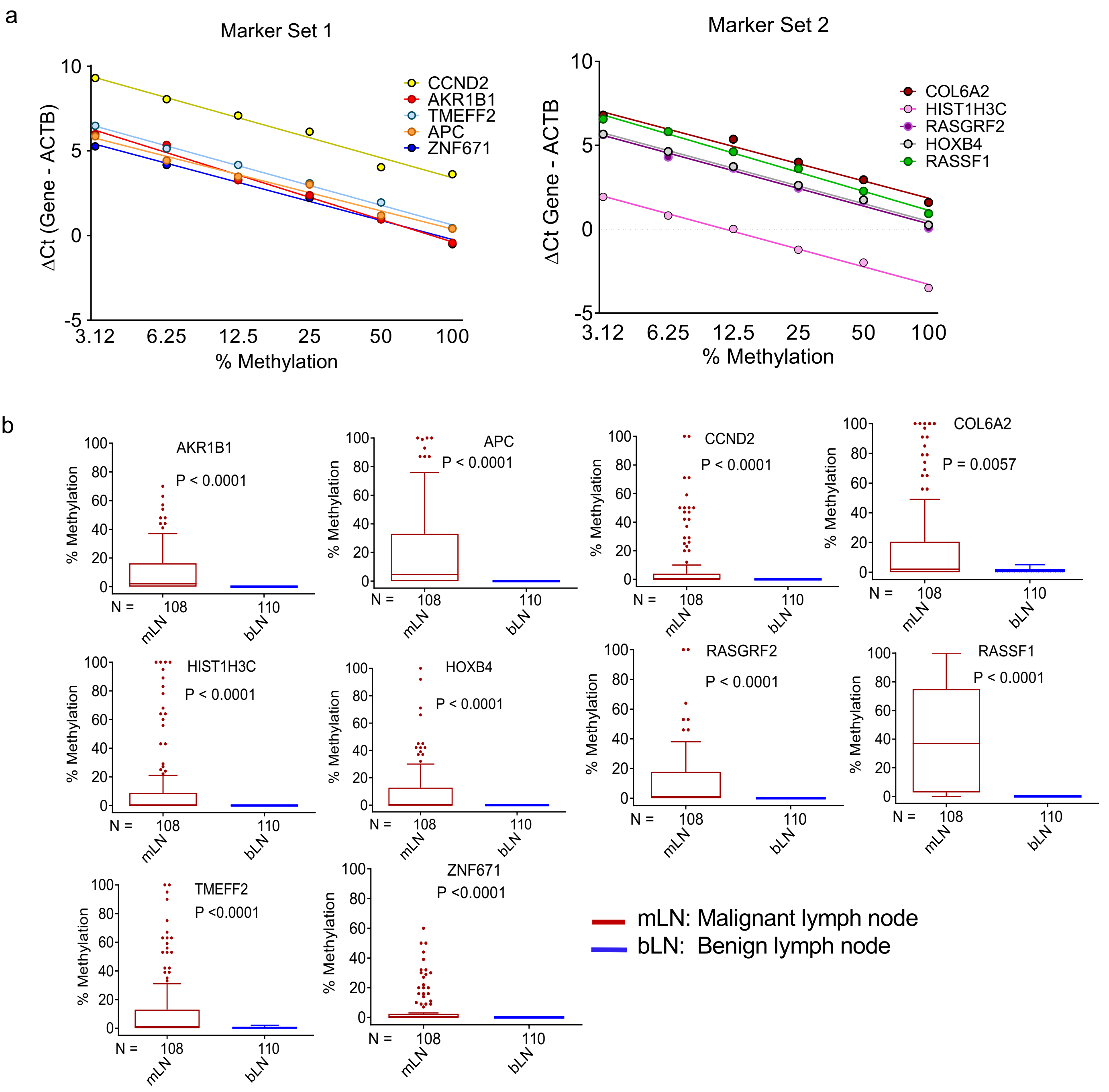

Supplementary Fig. 1.

a **Assay linearity and dynamic range of BCDA**. For assessing methylation in the FNA samples, standard curves were prepared. As published before (39) DNA aliquots were prepared from CAMA-1 cell line (100% methylated for 10 targets) and human sperm (0% methylated) and adjusted to a dilution at which 1 µl DNA = Ct 25 (cycle threshold) when assessed by BCDA. A dilution curve ranging from 100% - 3.12% methylation was prepared. Plotted is the average ΔCt (Ct Gene - Ct ACTB; linear Y-axis) at dilution (log2 X-axis), using nonlinear fit regression and 6 replicates per point for each of Gene Set 1 and Set 2, as indicated.

b **Performance of the 10 individual gene markers in the prospective study.** Box-Whiskers plots (Tukey method) depict the percent methylation (Y-axis) for each of the 10-gene markers in FNA from lymph nodes collected intraoperatively from patients with breast cancer. LNs were designated malignant or benign based upon histopathologic diagnosis. Mann-Whitney P-values are indicated. For the box plot, the whiskers are Tukey, the box is 25 and 75 percentiles, and the center bar is the median. Sample number (N) is shown below the X axis. mLN: malignant lymph nodes, bLN: benign lymph nodes.

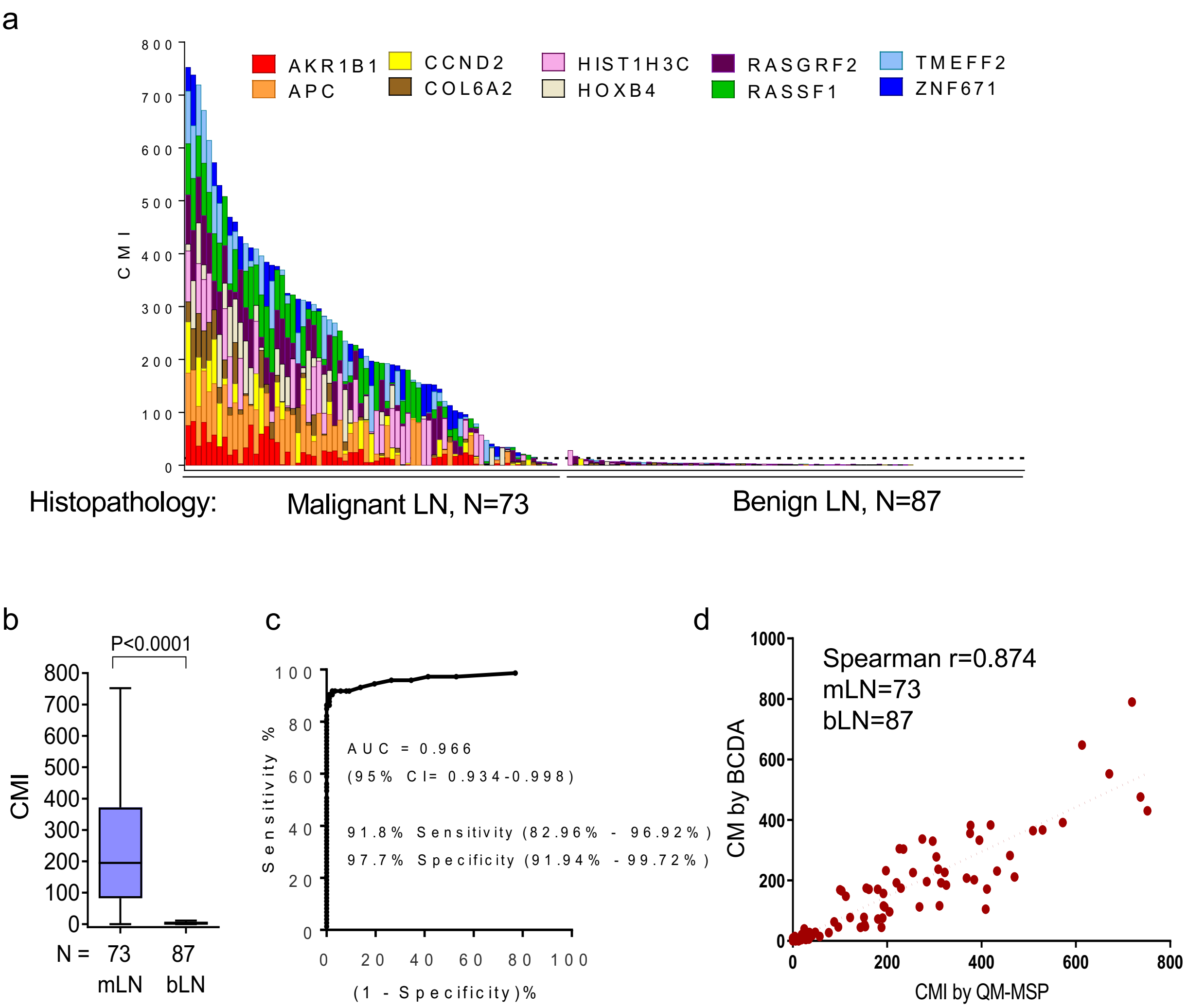

Supplementary Fig. 2 Assessment of gene methylation in lymph node-FNA by QM-MSP and comparison of its performance to the GeneXpert Prototype BCDA.

a For a subset of LN- FNA samples analyzed using by BCDA in the prospective study, QM-MSP was also performed. Cumulative methylation index for the 10-gene panel by QM-MSP analysis of the FNA from both malignant (N=73) and benign (N=87) LNs is shown in the histogram, and compared to histopathology.

b Box plots of CMI in malignant (mLN) and benign (bLN) lymph nodes are presented, which show a highly significant difference between the two groups. Mann-Whitney analysis p value: P<0.0001. For the box plot, the whiskers are Tukey, the box is 25 and 75 percentiles, and the center bar is the median.

c Receiver Operator Characteristics plot shows the discriminatory power of the 10-gene marker panel, with area under the ROC curve (AUC), sensitivity and specificity achieved by the QM-MSP assay.

d Comparison of CM of LN-FNA by BCDA versus CMI of the same LN-FNA by QM-MSP for the ten-gene panel shows a high level of correlation. Spearman correlation r=0.874; P=<0.00011.

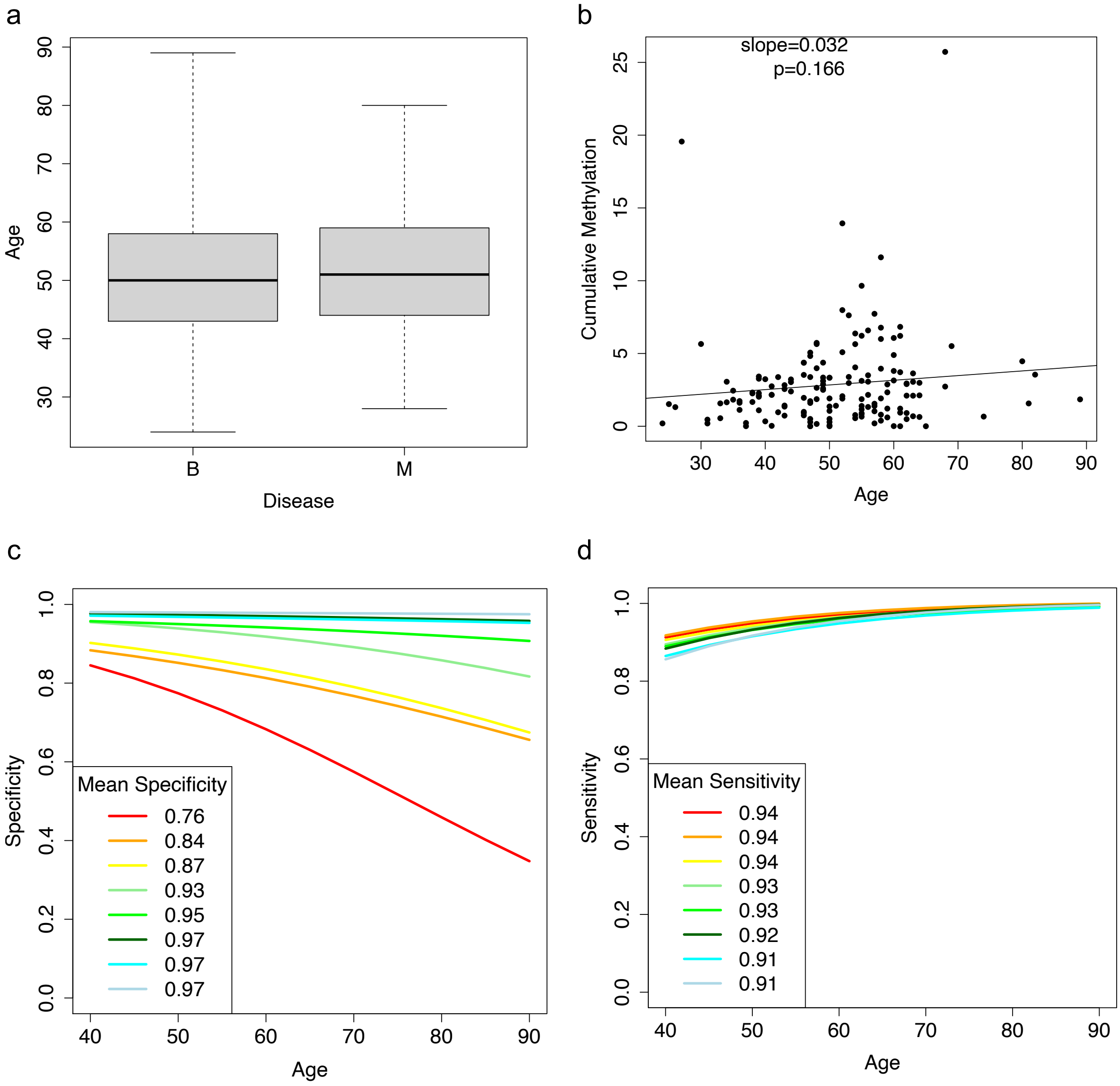

Supplementary Fig. 3 Effect of Patient Age on Methylation

Data from both the prospective and pilot clinical validation studies on malignant (N=180) and benign (N=156) LN-FNAs from China and the U.S were pooled to analyze effect of age on methylation in the LN-FNA. Age information for 6 benign samples from China were missing.

a Patients with benign and malignant disease are well matched by age. Box-plot shows no significant difference in age between patients with benign (B) and malignant (M) disease in the study. Mann-Whitney P-value: 0.292. For the box plot, the whiskers are Tukey, the box is 25 and 75 percentiles, and the center bar is the median.

b A linear regression model of CM by the BCDA as a function of age, fit on benign/normal samples (Panel **a**) demonstrates a modest, non-statistically significant increase in methylation with greater age. The fitted model is shown as a solid line. The slope of 0.032 for age corresponds with a doubling of CM from over 31 years.

c Logistic regression models show how age affects classification accuracy in benign samples. Individual lines correspond to possible thresholds for distinguishing between cases and controls. For the lower thresholds, corresponding to overall specificities below 90%, older patients with benign disease are likely to be misclassified. At the higher thresholds we are using in this paper, the misclassification rate varies much less by age as shown by the blue/green lines.

d The corresponding model for tumor samples shows a decrease in the risk of false negatives for older patients, although the effects are very small. The corresponding model for tumor samples shows a decrease in the risk of false negatives for older patients, although the effects are very small.

bLN, benign lymph node; mLN, malignant lymph node; BC, breast cancer; ER, estrogen receptor; PR, progesterone receptor; HER2, human epidermal growth factor receptor 2; TNBC, triple negative breast cancer.

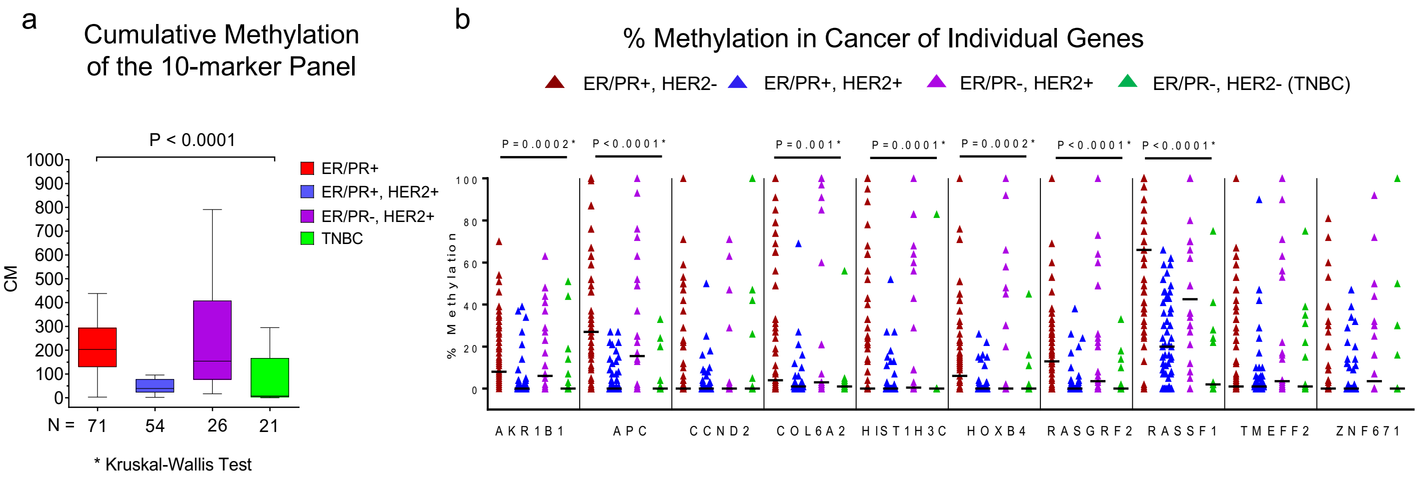

Supplementary Fig. 4 Gene Methylation assessed by BCDA in malignant LN-FNA samples by ER/PR/HER2 status of the primary tumor.

a Significant difference in cumulative gene methylation in the 10-marker panel is observed in the LN-FNA from patients in the prospective study with the four immunohistochemical subtypes of breast cancer (P= 0.003 by Kruskal-Wallis Test). For the box plot, the whiskers are Tukey, the box is 25 and 75 percentiles, and the center bar is the median.

b DNA methylation levels in each of the ten genes in LN-FNA from patients bearing ER/PR+, HER2- (red), ER/PR+, HER2+ (blue), ER/PR–, HER+ (fuchsia), ER/PR-, HER2- (green) tumors. (Also refer to Supplementary Tables S2a, 2b). Significant differences between subtypes in shown for 7/10 gene markers.

bLN, benign lymph node; mLN, malignant lymph node; BC, breast cancer; ER, estrogen receptor; PR, progesterone receptor; HER2, human epidermal growth factor receptor 2; TNBC, triple negative breast cancer.

a

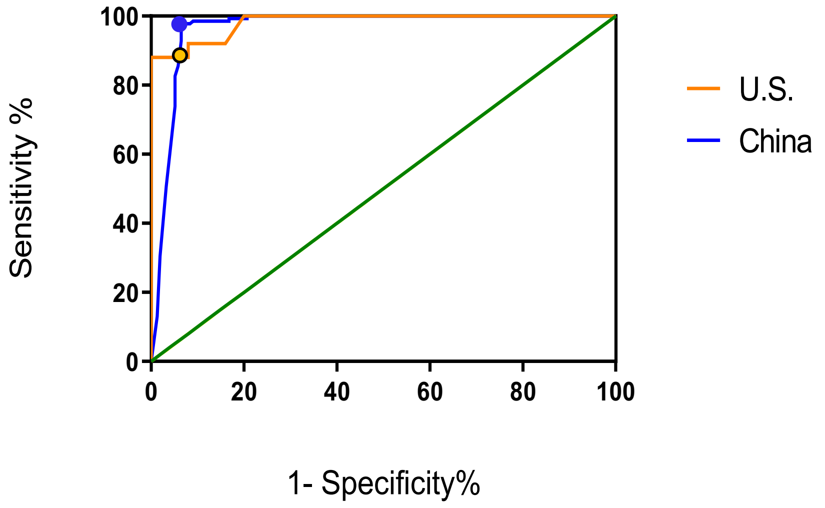

b

The BCDA system: Sensitivity/Specificity of detection by Region

Region	LN samples	Sensitivity*	Specificity*	AUC (95%CI)
U.S.	25 bLN, 25 mLN	88.0% (68.8%-97.5%)	92.0% (74.0%-99.0%)	0.982 (0.956-1.009)
China	138 bLN, 155 mLN	97.8% (93.8%-99.6%)	92.9% (87.7%-96.4%)	0.964 (0.940-0.988)

*Above 8.5 cumulative methylation units.
Abbreviation: bLN, benign lymph node; mLN, malignant lymph node. FNAs

Supplementary Fig. 5 The 10-gene panel examined by the BCDA shows similar level of performance in LN-FNA samples from the U.S. and China.

a Performance of the 10-gene panel (as measured by area under the ROC curve) was assessed for all LN-FNAs from the U.S. and China from both prospective and pilot clinical validation studies by BCDA.

b Sensitivity, specificity and AUC values with 95% CI are shown for samples from the U.S and China in the Table. Colored circles denote the threshold set at 8.5 CM units set in the prospective study.

bLN: benign lymph nodes, mLN: malignant lymph nodes

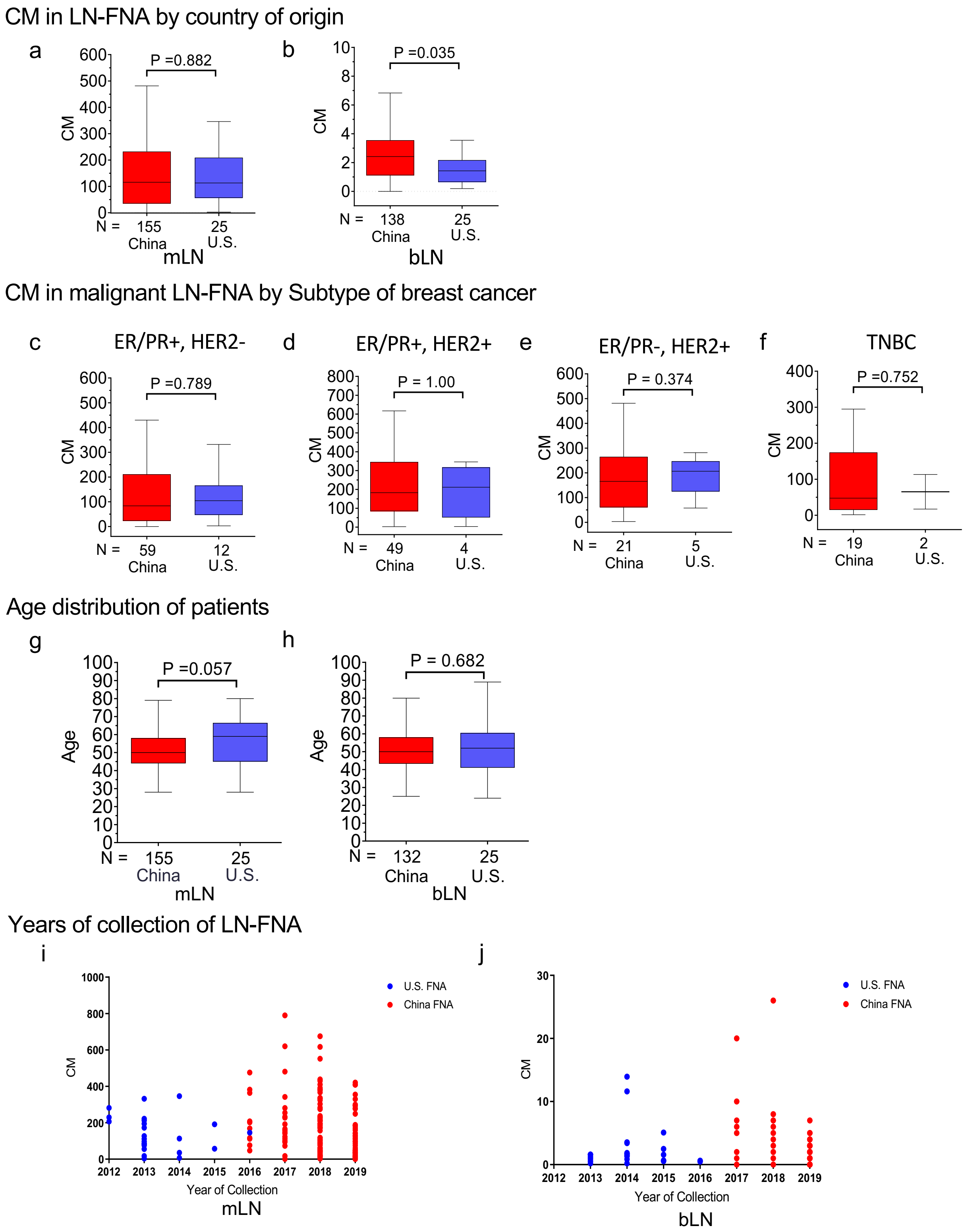

Supplementary Fig. 6 Factors that may contribute to sensitivity and specificity of BCDA in LN-FNAs from China and the U.S.

Data from both prospective and pilot clinical validation studies (China: 155 malignant, 138 benign; US: 25 malignant, 25 benign) on LN-FNA were analyzed for the following comparisons.

a-b: Box plots comparing CM in malignant (**a**) and benign (**b**) LN-FNA from the U.S and China are shown. Mann-Whitney P values for CM in malignant LN-FNAs in samples from either country were not significant (P=0.882), but significant difference was observed in the benign LN-FNAs (P=0.035). For the box plots, the whiskers are Tukey, the box is 25 and 75 percentiles, and the center bar is the median.

c-f: Box plots comparing CM in malignant and benign LN-FNA from the U.S. and China in various pathological subtypes of breast cancer are shown. No difference in methylation was observed as seen by Mann-Whitney P values. For the box plots, the whiskers are Tukey, the box is 25 and 75 percentiles, and the center bar is the median.

g-h: While study participants from China with malignant LNs were slightly younger than those from the U.S., this difference did not reach statistical significance (P=0.057) (**g**). There was no significant difference in age of the patients who contributed benign FNA samples (**h**). Age information was missing for 6/138 benign samples. For the box plots, the whiskers are Tukey, the box is 25 and 75 percentiles, and the center bar is the median.

i-j: The length of storage of the FNA samples prior to analysis by BCDA was investigated. LN-FNA samples from the U.S. were stored for significantly longer time and were collected from years 2012-2016 (**i**), while samples from China were from 2016-2019 (**j**).

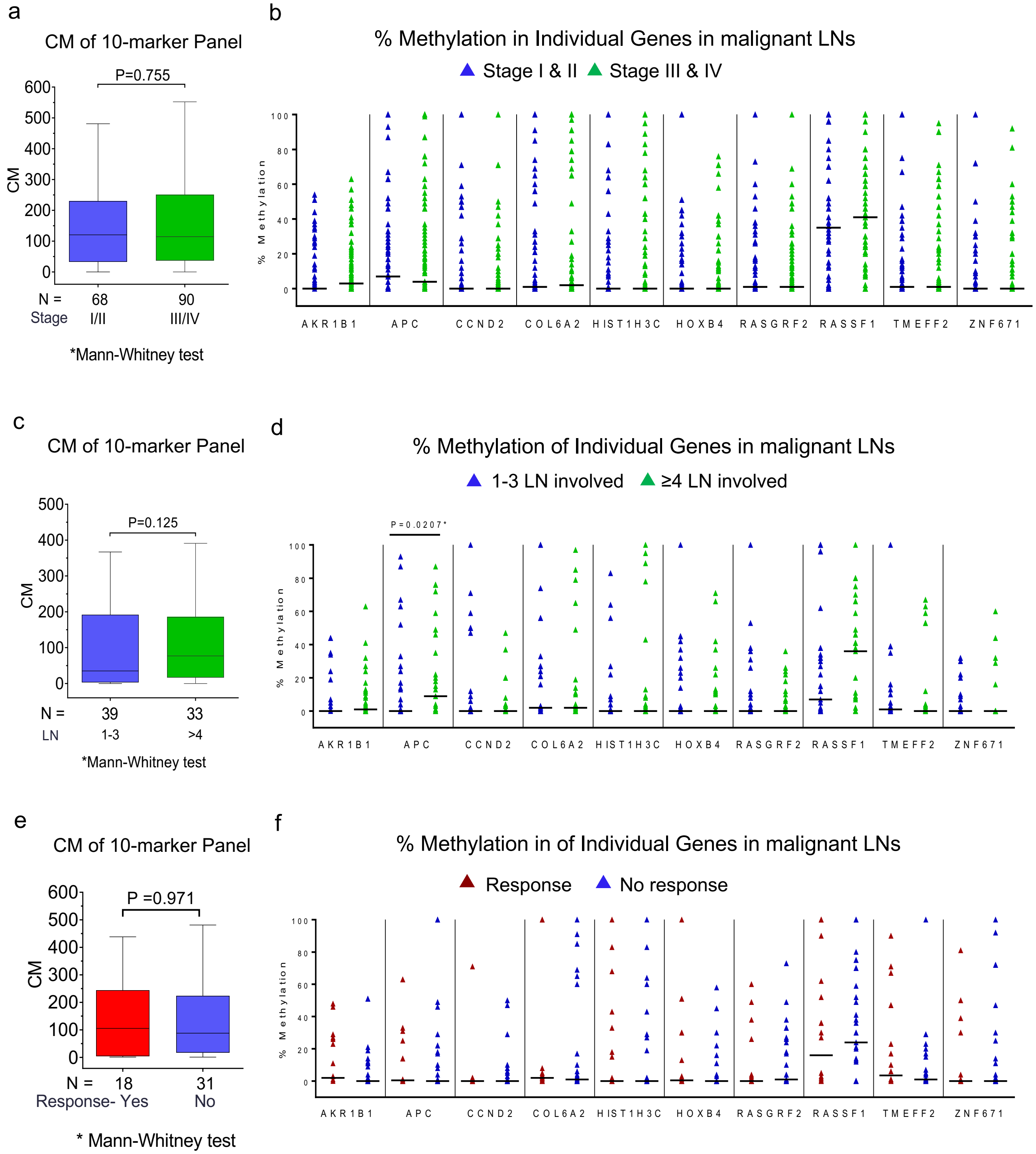

Supplementary Fig. 7 Performance of the 10 individual gene markers by BCDA in LN- FNA by clinical stage, number of involved lymph node, and response to neoadjuvant chemotherapy (NAC).

a, b Correlation of cumulative (**a**) and single gene methylation (**b**) in FNA of malignant lymph node as determined by BCDA with clinical stage of breast cancer. DNA methylation in stage I & II (blue), stage III & IV (green) using pooled samples (N= 158) from prospective (N=99/108) and pilot clinical validation (N= 58, China, 34/47; U.S, 24/25) studies. For comparison between two groups the Mann-Whitney test, unadjusted P-values are indicated (*). For the box plot, the whiskers are Tukey, the box is 25 and 75 percentiles, and the center bar is the median. Sample number (N) is shown below the X-axis.

c, d Correlation of cumulative (**c**) and single gene (**d**) methylation in LN-FNA of malignant tested lymph node with number of involved lymph node in the prospective study. Box-Whiskers plots, the whiskers are Tukey, the box is 25 and 75 percentiles, and the center bar is the median, and scatter plots depict the percent methylation (Y-axis) for each of the 10-gene markers in LN-FNA specimens obtained from the test lymph node. DNA methylation in 1-3 lymph nodes involved (blue), and ≥ 4 lymph nodes involved (green).

e, f Correlation of cumulative (**e**) and single gene (**f**) methylation in LN-FNA of malignant lymph node with response to NAC for a subset (49/78 patients) of the pilot clinical validation study. Box-Whiskers plots, the whiskers are Tukey, the box is 25 and 75 percentiles, and the center bar is the median, and scatter plots depict the percent methylation (Y-axis) for each of the 10-gene markers in FNA specimens obtained from lymph node. DNA methylation in lymph node in patients who responded to NAC (red), and who did not respond to NAC (blue). LN, lymph node; BC, breast cancer; NAC, neoadjuvant chemotherapy; FNA, fine needle aspiration.
